# *jaw-1D*: a gain-of-function mutation responsive to paramutation-like induction of epigenetic silencing

**DOI:** 10.1093/jxb/ery365

**Published:** 2018-10-20

**Authors:** Wen Jiang, Zhongfei Li, Xiaozhen Yao, Binglian Zheng, Wen-Hui Shen, Aiwu Dong

**Affiliations:** 1 State Key Laboratory of Genetic Engineering, Collaborative Innovation Center for Genetics and Development, International Associated Laboratory of CNRS-Fudan-HUNAU on Plant Epigenome Research, Institute of Plant Biology, School of Life Sciences, Fudan University, Shanghai, China; 2 College of Life and Environment Sciences, Shanghai Normal University, Shanghai, PR China; 3 State Key Laboratory of Genetic Engineering, Collaborative Innovation Center of Genetics and Development, Institute of Plant Biology, School of Life Sciences, Fudan University, Shanghai, PR China; 4 Université de Strasbourg, CNRS, Strasbourg, France

**Keywords:** *Arabidopsis thaliana*, DNA methylation, epigenetic silencing, histone methylation, *jaw-1D*, paramutation

## Abstract

The *Arabidopsis thaliana* gain-of-function T-DNA insertion mutant *jaw-1D* produces miR319A, a microRNA that represses genes encoding CIN-like TEOSINTE BRANCHED1/CYCLOIDEA/PROLIFERATING CELL FACTORs (TCPs), a family of transcription factors that play key roles in leaf morphogenesis. In this study, we show that *jaw-1D* is responsive to paramutation-like epigenetic silencing. A genetic cross of *jaw-1D* with the polycomb gene mutant *curly leaf-29* (*clf-29*) leads to attenuation of the *jaw-1D* mutant plant phenotype. This induced mutation, jaw-1D*, was associated with down-regulation of miR319A, was heritable independently from *clf-29*, and displayed paramutation-like non-Mendelian inheritance. Down-regulation of miR319A in jaw-1D* was linked to elevated levels of histone H3 lysine 9 dimethylation and DNA methylation at the CaMV35S enhancer located within the activation-tagging T-DNA of the *jaw-1D* locus. Examination of 21 independent T-DNA insertion mutant lines revealed that 11 could attenuate the *jaw-1D* mutant phenotype in a similar way to the paramutation induced by *clf-29*. These paramutagenic mutant lines shared the common feature that their T-DNA insertion was present as multi-copy tandem repeats and contained high levels of CG and CHG methylation. Our results provide important insights into paramutation-like epigenetic silencing, and caution against the use of *jaw-1D* in genetic interaction studies.

## Teaser

An Arabidopsis gain-of-function T-DNA insertion mutant, *jaw-1D*, has an unexpected paramutation phenomenon caused by transgenic SALK mutant lines with multi-copy tandem T-DNA repeats.

## Introduction

MicroRNAs (miRNAs) are non-coding RNAs about 21–24 nucleotides in length that are important regulators of gene expression in both animals and plants. In *Arabidopsis thaliana*, miRNAs are involved in a wide range of biological processes, including meristem identity, leaf polarity, flowering patterning, signaling pathways, and responses to environmental stress (Xie *et al.*, 2015; Yu *et al.*, 2017). For miRNA synthesis, an *miR* gene is first transcribed into primary miRNA (pri-miRNA) by RNA polymerase II. pri-miRNA is then processed into pre-miRNA by DICER-LIKE 1 (DCL1) assisted by HYPONASTIC LEAVES and SERRATE proteins. The pre-miRNA is further processed into a miRNA/miRNA* duplex consisting of the guide-strand miRNA and the passenger-strand miRNA*. Lastly, one strand of the duplex is incorporated into an ARGONAUTE (AGO) protein to carry out downstream functions, such as slicing/degradation and/or translational repression of target RNA molecules (Xie *et al.*, 2015; Yu *et al.*, 2017).

Arabidopsis miR319A was initially identified through the analysis of *jagged and wavy Dominant* (*jaw-D*) mutant alleles in a large-scale T-DNA insertion activation tagging gain-of-function mutagenesis (Weigel *et al.*, 2000; Palatnik *et al.*, 2003). *jaw-D* mutant plants exhibit a curly, uneven shape and serrated leaves because of miR319A overexpression. A subset of Teosinte branched1/Cycloidea/Proliferating cell factor (TCP) family transcription factors has been characterized as the targets of miR319A (Palatnik *et al.*, 2003). TCP proteins are plant-specific transcription factors that share a conserved basic helix–loop–helix DNA-binding domain, termed the TCP domain (Cubas *et al.*, 1999). In Arabidopsis, the TCP family consists of 24 members with 13 in the class-I group and 11 in the class-II group, based on the conservation and organization of the TCP domain. Class-II TCPs are further classified into the subgroups CIN-like (eight members) and CYC/TB1-like (three members) (Martín-Trillo and Cubas, 2010). Five CIN-like TCP genes (*TCP2*, *TCP3*, *TCP4*, *TCP10*, and *TCP24*) have been shown to be miR319A targets, and their transcript levels are dramatically reduced in *jaw-D* because of the ectopic expression of miR319A (Palatnik *et al.*, 2003). Because single *tcp* loss-of-function mutants only have mild phenotypes through functional redundancy of different *TCP*s, *jaw-D* has been widely used to study the functions of CIN-like TCPs and their genetic interactions with other factors in the regulation of plant development (Palatnik *et al.*, 2003; Schommer *et al.*, 2008; Nag *et al.*, 2009; Liu *et al.*, 2011; Danisman *et al.*, 2012; Tao *et al.*, 2013; Zhang *et al.*, 2017). Our own previous work has demonstrated that miR319A-regulated TCPs physically interact with ASYMMETRIC LEAVES2 (AS2), and showed that the *as2-1 jaw-1D* double-mutant has enhanced leaf developmental defects (Li *et al.*, 2012).

In contrast to these examples that successfully demonstrate the use of *jaw-D* as a TCP-knockdown mutant, we show here that *jaw-1D* is responsive to paramutation-like induction of epigenetic silencing, which prevents its use as a bona fide TCP-knockdown tool. Paramutation is an epigenetic phenomenon first described in depth in maize (Brink, 1956) and later found to be present in many multicellular organisms (Hövel *et al.*, 2015; Hollick, 2017; Pilu, 2015). It describes the heritable *trans*-interactions that occur between two homologous alleles that exhibit different transcriptional activities. Usually, the weakly expressed allele (the paramutagenic allele) can transform the highly expressed allele (the paramutable allele) into a new paramutagenic allele. Importantly, the newly transformed/paramutated allele is mitotically and meiotically stable and can induce paramutable-to-paramutagenic allele transformation.

The emerging molecular mechanisms of paramutation implicate self-reinforcing feedback loops carried out by small RNA biogenesis and chromatin modifications (Hollick, 2017). While animals deploy the PIWI-interacting RNA pathway together with repressive histone modifications, plants have evolved the RNA-dependent DNA methylation (RdDM) pathway. In this RdDM model, siRNA (24 nt in length) produced by a paramutagenic allele reinforces the silenced state of the paramutagenic allele and initiates silencing of the corresponding paramutable allele via cytosine (C) methylation in all different DNA sequence contexts (CG, CHG, and CHH; H=A, T, or C) together with methylation of histone-3 lysine-9 (H3K9) and H3K27 (Hövel *et al.*, 2015; Hollick, 2017). Using a multi-copy *pRD29A-LUC* transgene as a paramutable allele in Arabidopsis, it has been demonstrated that numerous factors involved in RdDM, CG/CHG methylation, or histone modifications are required for paramutation-like silencing of the *pRD29A-LUC* transgene (Zheng *et al.*, 2015). Nevertheless, the paramutation phenomena identified thus far are limited to a relatively small number of examples, and detailed case-by-case examination has unraveled differences regarding the frequency of paramutation, the heritability and stability of the paramutated state, as well as the occurrence of spontaneous and secondary paramutations (Hövel *et al.*, 2015). The identification of additional paramutation examples is likely to help in understanding the conservation and diversity of the molecular mechanisms underlying paramutation.

In the present study, we report the identification of *jaw-1D* as a novel paramutable allele. We provide evidence that the heterochromatin mark di-methylation of histone-3 lysine-9 (H3K9me2) and DNA methylations are associated with *jaw-1D* paramutation. Furthermore, we show that numerous T-DNA insertion mutants of various genes are paramutagenic to *jaw-1D*, which not only provides useful information about *cis*-elements involved in paramutation but also cautions against the use of *jaw-1D* in genetic interaction studies where combined mutants are generated.

## Materials and methods

### Plant material and growth conditions

All Arabidopsis alleles used in this work were derived from the Columbia ecotype (Col-0). The following SALK line mutants were obtained from the Arabidopsis Biological Resource Center (www.arabidopsis.org): *jaw-1D* (CS6948), *clf-29* (SALK_021003), *dcl2-1* (SALK_064627), *sdg8-1* (SALK_065480), *sde4-3* (SALK_128428), *mapkkk21* (SALK_018714C), *at1g19340-1* (SALK_070222), *at1g19340-2* (SALK_133379), *at1g19340-3* (SALK_008485), *chr24-1* (SALK_152488), *atx1-2* (SALK_149002), *chr19* (SALK_069014), *nap1;2-1* (SALK_131746), *ago6-2* (SALK_031553C), *hyl1-2* (SALK_064863), *smc6b-1* (SALK_101968C), *arid1-1* (SALK_047099), *nrpe1-12* (SALK_033852), *mpk17* (SALK_020801C), *nrpd2a-2* (SALK_046208), *hdt3* (SALK_039784), and *at5g07810* (SALK_127508). For seed production, genetic interaction, and phenotype and genotyping analyses, plants were grown in soil in greenhouses under a 16/8 h light/dark photoperiod (light intensity 100~120 μmol photons m^–2^ s^–1^ at leaf level). For all other experiments, plants were cultured *in vitro* on agar-solidified Murashige and Skoog medium M0255 (Duchefa Biochemie) with 0.9% sucrose at 22 °C under a 16/8 h light/dark photoperiod (light intensity ~100 μmol photons m^–2^ s^–1^ at leaf level). For the kanamycin (Km) resistance test, the medium was supplemented with 50 mg l^–1^ Km.

### Gene expression analysis

Total RNA was prepared from 2-week-old seedlings using TRI Reagent (Invitrogen) according to the manufacturer’s instructions. Reverse transcription was performed using standard procedures with Improm-II reverse transcriptase (Promega). PCR amplifications from the cDNA template were performed using gene-specific primers (Supplementary Table S1 at *JXB* online). *UBQ10* was used as the reference gene in normalization.

### Chromatin immunoprecipitation assays

Chromatin immunoprecipitation (ChIP) assays were performed on 2-week-old seedlings according to a previously described method (Saleh *et al.*, 2008) using the following antibodies: anti-trimethyl-H3K27 (07-449; Millipore), anti-trimethyl-H3K4 (07-473; Millipore), and anti-dimethyl-H3K9 (ab1220; Abcam). The gene-specific primers used in PCR are listed in Supplementary Table S1.

### Bisulfite genomic sequencing

Genomic DNA was extracted from 2-week-old seedlings using the standard CTAB protocol (Clarke, 2009). A total of 2 μg of RNase-treated genomic DNA was subjected to bisulfite conversion using an EpiTect Bisulfite Kit (Qiagen) according to the manufacturer’s instructions. Converted DNA then underwent PCR using primers listed in Supplementary Table S1. The PCR products were cloned into the pGEM-T Easy vector (Promega). Over 10 independent clones were sequenced and analysed for DNA methylation.

### T-DNA copy number analysis

T-DNA copy numbers were estimated by real-time quantitative (q)PCR as previously described (Ingham *et al.*, 2001; Bubner and Baldwin, 2004). Genomic DNA was extracted from 2-week-old seedlings using the standard CTAB protocol (Clarke, 2009). *TA3* was used as the endogenous control gene in normalization. The *nrpe1-12* mutant was chosen as the calibrator because it had the smallest Δ*C*_T_ (*C*_T(TA3)_–*C*_T(35S)_) in CaMV35S amplification. The primers used in qPCR are listed in Supplementary Table S1.

## Results

### Silencing of *jaw-1D* by *clf-29*

**Fig. 1.**
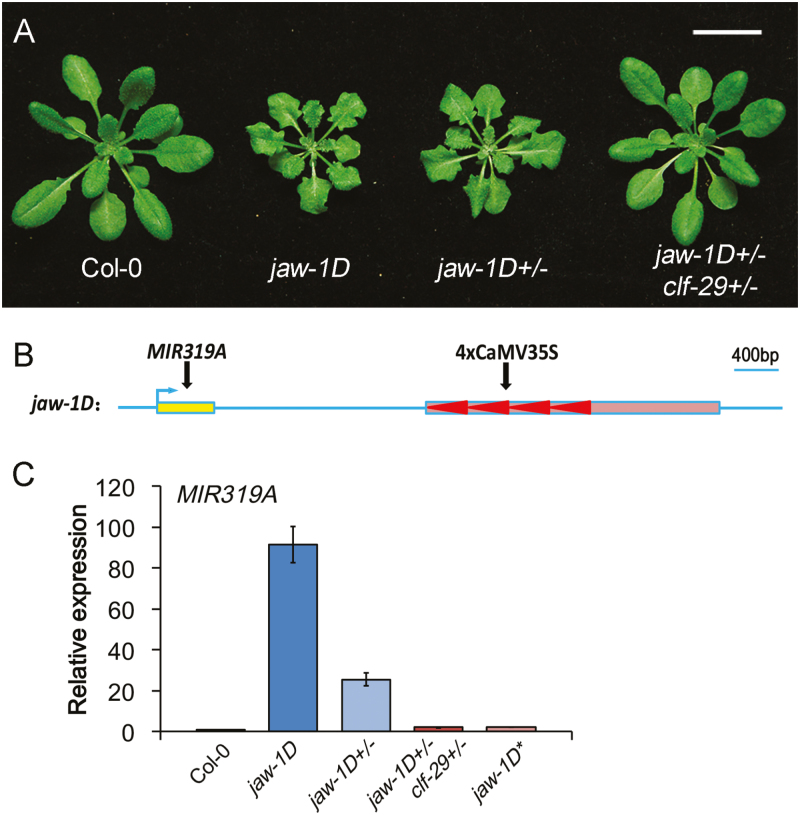
*jaw-1D* is silenced by *clf-29*. (A) Phenotype comparison of 22-d-old plants of wild-type Col-0 and mutants. The scale bars is 2 cm. (B) Diagram of the *jaw-1D* mutant locus structure. The mi*R319A* gene and the 4xCaMV35S enhancer (arrowheads) within the T-DNA (box) are indicated. (C) qRT-PCR analysis of transcription levels of *miR319A* pri-miRNA in Col-0 and mutants. Expression levels of *miR319A* were normalized to *UBQ10*. Relative values in the mutants compared with the wild-type Col-0 (set as 1) are shown as means (±SD) from three biological repeats.

Our previous studies showed that both CIN-like TCPs and polycomb group (PcG) proteins are involved in AS1/AS2-mediated repression of Class-I *KNOX* genes (Li *et al.*, 2012, 2016). To further analyse the functional relationship between CIN-like TCPs and PcG proteins, we crossed *jaw-1D* with *clf-29*, a T-DNA insertion mutant with the *CURLY LEAF* gene coding for H3K27-methyltransferase (Xu and Shen, 2008). Because *jaw-1D* represents a gain-of-function mutation whereas *clf-29* is a recessive loss-of-function mutant, plants from the F1 generation of this cross (*jaw-1D+/– clf-29+/–*) were expected to exhibit a phenotype similar to heterozygous *jaw-1D* (*jaw-1D+/–*). Surprisingly, however, while *jaw-1D+/–* plants displayed a phenotype largely similar to *jaw-1D* with jagged and serrated leaves, *jaw-1D+/– clf-29+/–* plants showed a wild-type phenotype (Fig. 1A).

Quadruple repeats of the CaMV35S enhancer (4xCaMV35S), which ectopically activate *miR319A* expression (Palatnik *et al.*, 2003), were inserted downstream of the *miR319A* locus in *jaw-1D* (Fig. 1B). To identify the molecular mechanisms underlying the suppressed phenotype of *jaw-1D+/– clf-29+/–*, we analysed transcript levels of *miR319A* in rosette leaves by reverse-transcription qPCR. *miR319A* transcript levels were increased about 90-fold in *jaw-1D* and by over 20-fold in *jaw-1D+/–* but were almost unchanged in *jaw-1D+/– clf-29+/–* compared with the Col-0 wild-type control (Fig. 1C). These results indicated that the overexpression of *miR319A* in *jaw-1D+/–* was sufficient to induce the mutant plant phenotype, and that the wild-type phenotype of *jaw-1D+/– clf-29+/–* was associated with the attenuation of *miR319A* overexpression.

### The silencing of *jaw-1D* is inheritable and paramutagenic

**Fig. 2.**
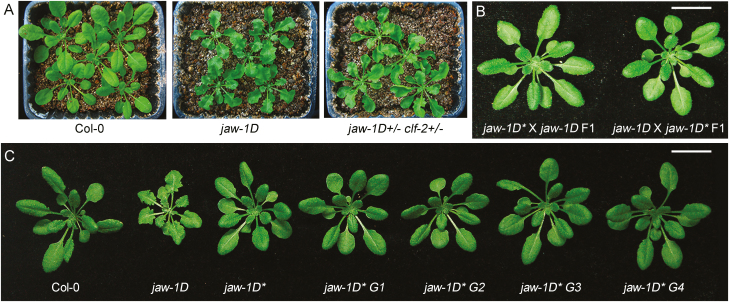
The silencing of *jaw-1D* is inheritable and paramutagenic. (A) Phenotypes of 22-d-old plants of wild-type Col-0 and mutants. The pot size is 9 × 8 cm. (B) Representative 22-d-old plants derived from reciprocal crosses between *jaw-1D* and jaw-1D*. The scale bar is 2 cm. (C) Representative 22-d-old plants of Col-0, *jaw-1D*, jaw-1D*, and successive next generations of jaw-1D* (G1–G4. *The* scale bar is 2 cm.

Because PcG proteins are generally known as repressors of transcription, the *miR319A* suppression observed in *jaw-1D+/– clf-29+/–* was unlikely to be linked with reduced CLF activity. Nevertheless, we directly examined this by crossing *jaw-1D* with *clf-2*, a transposon insertion mutant of *CLF* (Goodrich *et al.*, 1997). In contrast to *jaw-1D+/– clf-29+/–*, all *jaw-1D+/– clf-2+/–* plants displayed a phenotype similar to *jaw-1D* (Fig. 2A), indicating that *clf-2* did not induce silencing of *jaw-1D*. Next, we analysed the progeny of *jaw-1D+/– clf-29+/–* to investigate whether the silencing of *jaw-1D* was released in the next generation. In contrast to the expectation from classical genetic segregation, none of the individual plants from a selfing population of *jaw-1D+/– clf-29+/–* (over 80 individual plants examined) displayed a *jaw-1D*-like phenotype. The progeny plants with a *jaw-1D* genotype displayed a phenotype similar to wild-type Col-0, and we refer to them hereafter as jaw-1D* plants. We also tested the interaction between jaw-1D* and *jaw-1D* using reciprocal crosses. All F1 plants behaved like jaw-1D* and showed a wild-type phenotype (Fig. 2B). To check the genetic inheritance of jaw-1D*, we examined plant phenotypes of the next four successive selfing-generations of jaw-1D* (G1 to G4). As shown in Fig. 2C, plants from all the generations examined showed a wild-type phenotype, indicating stable inheritance of jaw-1D*. Taken together, our data indicate that *jaw-1D* is responsive to paramutation-like silencing, such that the silencing of *jaw-1D* (jaw-1D*) induced by *clf-29* is inheritable independently from *clf-29*, and that jaw-1D* is paramutagenic to *jaw-1D*.

### Analysis of epigenetic modifications associated with *jaw-1D* silencing

**Fig. 3.**
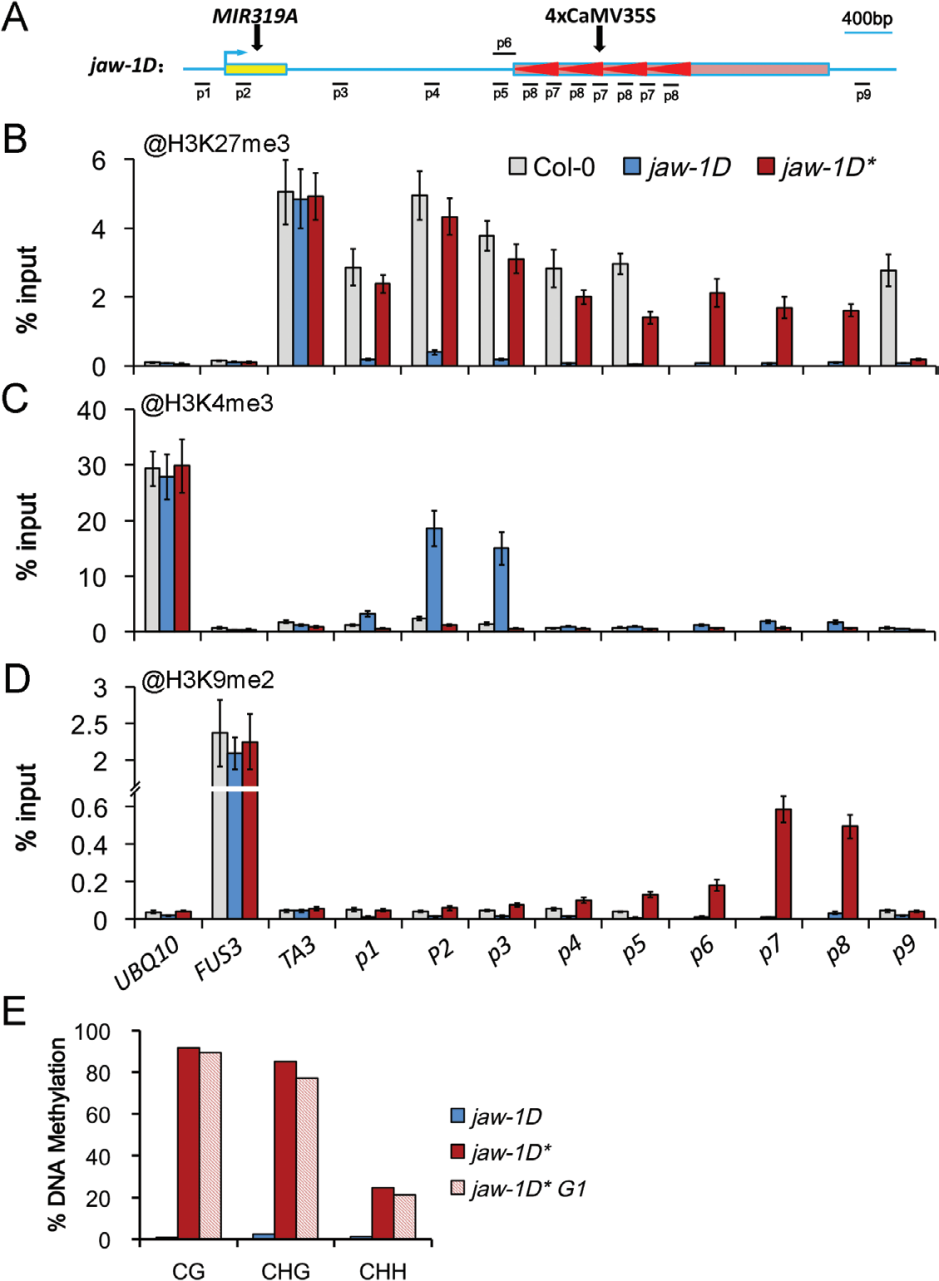
Analysis of epigenetic modifications at the *jaw-1D* locus. (A) Diagram of the regions analysed. The structure of the locus is the same as described in Fig. 1B. The numbers p1–p9 indicate the regions analysed. (B–D) ChIP–PCR analysis of H3K27me3, H3K4me3, and H3K9me2 levels in wild-type Col-0 and the mutants *jaw-1D* and jaw-1D*. The different regions of the *jaw-1D* locus and the controls *UBQ10*, *TA3*, and *FUS3* were analysed by real-time qPCR. Percentage-of-input values are shown as means (±SD) from three independent biological replicates. (E) Comparison of DNA methylation levels of CaMV35S in *jaw-1D*, jaw-1D*, and jaw-1D* G1 as examined by bisulfite sequencing. The CaMV35S sequence contained 12 CGs, eight CHGs, and 60 CHHs. Percentage-of-methylation values are shown based on a total of 11, 10, and 11 CaMV35S sequences cloned from *jaw-1D*, jaw-1D*, and jaw-1D* G1 samples, respectively. Detailed information can be found in Supplementary Table S2.

To gain information about the chromatin basis of *jaw-1D* silencing, we first analysed levels of different histone methylations. In Arabidopsis, H3K4me3 and H3K27me3 are known to be associated with transcription activation and transcription repression in euchromatin, respectively, whereas H3K9me2 marks stably silenced heterochromatin (Liu *et al.*, 2010). Consistently, our ChIP analysis revealed a high level of H3K4me3 at the actively transcribed gene *UBQ10*, a high level of H3K27me3 at the repressed gene *FUS3*, and a high level of H3K9me2 at the heterochromatic silenced transposon *TA3* (Fig. 3). It also showed no significant difference in *jaw-1D* and jaw-1D* heterochromatin compared with Col-0. We further analysed different regions covering *miR319A* to downstream of the T-DNA insertion site of *jaw-1D* (Fig. 3A). In Col-0, high levels of H3K27me3 were detected at various regions ranging from the promoter to at least 2000 bp downstream of the *miR319A* open reading frame (Fig. 3B), whereas H3K4me3 and H3K9me2 were barely detectable (Fig. 3C, D). In contrast, in *jaw-1D*, H3K27me3 levels were drastically reduced at all the regions examined, and high levels of H3K4me3 were detected at regions close to the *miR319A* transcription start site (Fig. 3B, C). These data were in agreement with the location of *miR319A* in euchromatin and indicated that active *miR319A* transcription was associated with removal of the repressive mark H3K27me3 and the deposition of H3K4me3 in *jaw-1D*. Most strikingly, in jaw-1D* the heterochromatin mark H3K9me2 was detected at 4xCaMV35S regions, and high levels of H3K27me3 were observed at both *miR319A* and 4xCaMV35S regions (Fig. 3). Thus, elevations of both H3K9me2 and H3K27me3 appeared to be associated with the stable silencing of *miR319A* in jaw-1D*. Region-9 downstream of the T-DNA insertion site did not show elevated H3K27me3 in jaw-1D* as in Col-0 (Fig. 3), suggesting that the H3K9me2-associated heterochromatinization may have caused H3K27me3 elevation at specific regions.

In Arabidopsis, H3K9me2 acts closely together with DNA methylation in heterochromatic silencing. The two types of modifications appear to be part of a reinforcing loop, in that H3K9 methylation recruits RdDM components and DNA methyltransferases, and H3K9-methyltransferases bind methylated DNA to favor H3K9 methylation (Law *et al.*, 2013; Johnson *et al.*, 2014; Liu *et al.*, 2014). We carried out bisulfite sequencing analysis to examine DNA cytosine methylation levels at the 4xCaMV35S enhancer in *jaw-1D* and jaw-1D*. About 92% of CG (*n*=120), 85% of CHG (*n*=80), and 25% of CHH (*n*=600) were methylated in jaw-1D* but were at almost undetectable levels in *jaw-1D* (Fig. 3E, Supplementary Table S2). The DNA methylation status was stably inherited because the plants of the next generation (jaw-1D* G1) showed similar levels of CG, CHG, and CHH methylation to the parental jaw-1D* plants (Fig. 3E, Supplementary Table S2). Taken together, we conclude that the silencing of *jaw-1D* is associated with both H3K9me2 and DNA methylation occurring at the 4xCaMV35S enhancer.

### Analysis of epigenetic modifications associated with *clf-29*

**Fig. 4.**
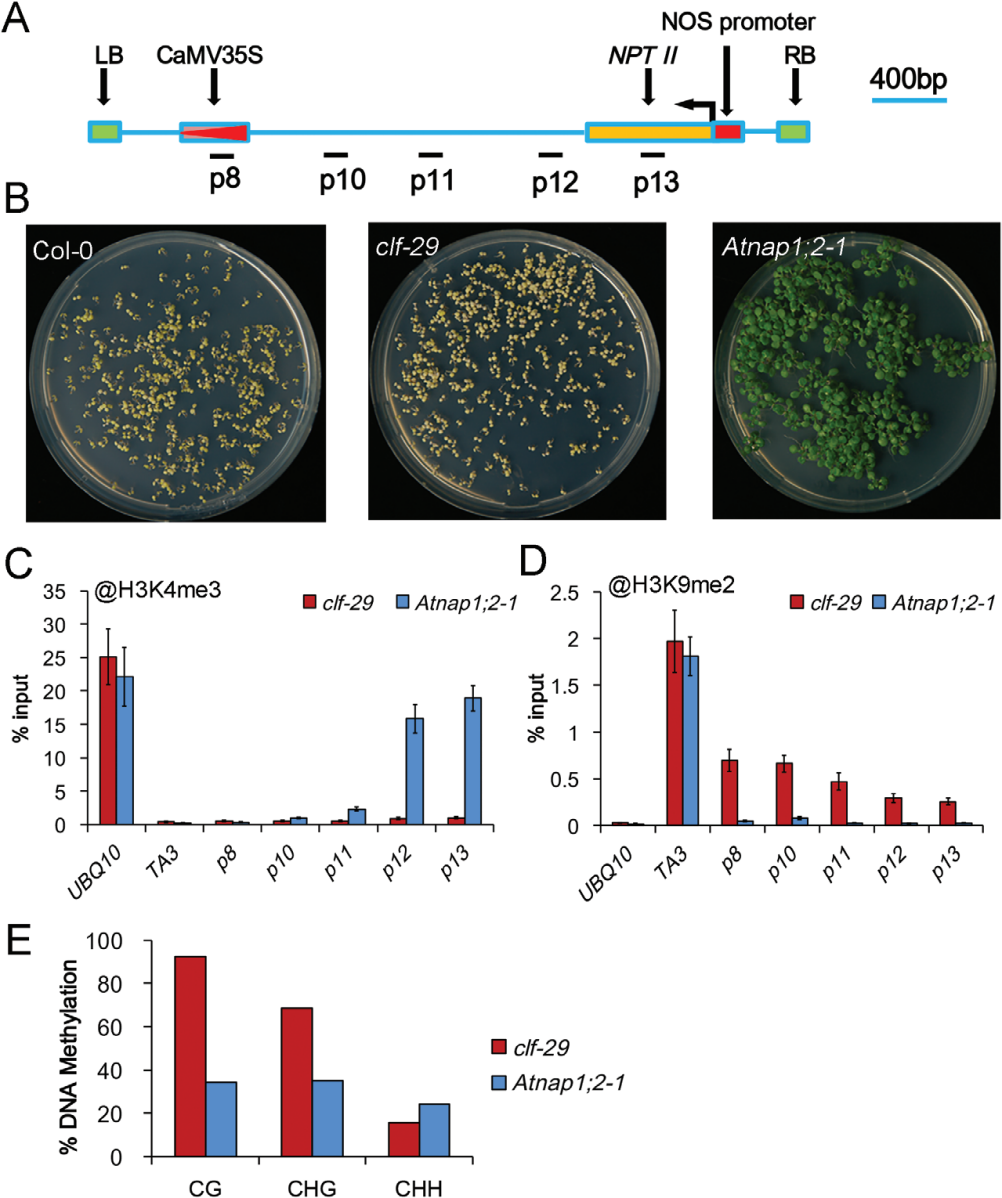
T-DNA in *clf-29* is highly heterochromatinized. (A) Diagram of structure and regions analysed of T-DNA in *clf-29*. The T-DNA left border (LB) and right border (RB), the kanamycin (Km)-resistance selection marker *NPTII* driven by the NOS promoter, and the CaMV35S region are indicated. The numbers p8–p13 indicate the regions analysed. (B) Representative plates of wild-type Col-0 and the mutants *clf-29* and *Atnap1;2-1* grown on medium containing Km. (C, D) ChIP–PCR analysis of H3K4me3 and H3K9me2 levels at T-DNA regions in *clf-29* and *Atnap1;2-1*. The different regions of T-DNA and controls *UBQ10* and *TA3* were analysed by real-time qPCR. Percentage-of-input values are shown as means (±SD) from three independent biological replicates. (E) Comparison of DNA methylation levels of CaMV35S in *clf-29* and *Atnap1;2-1* as examined by bisulfite sequencing. Percentage-of-methylation values are shown based on a total of 12 independent CaMV35S sequences cloned from each mutant sample. Detailed information can be found in Supplementary Table S2.

*jaw-1D* paramutation-like silencing was induced by the T-DNA insertion mutant *clf-29* but not by the transposon insertion mutant *clf-2*. To better understand paramutagenic *cis*-determinants, we analysed the conformation of the T-DNA structure in *clf-29*. We found that besides *NEOMYCIN PHOSPHOTRANSFERASE II* (*NPTII*) driven by the NOS promoter, which served as a selection marker of plants resistant to Km, T-DNA also carried a copy of CaMV35S (Fig. 4A). Considering that paramutation is induced by homolog *trans*-interactions (Hövel *et al.*, 2015), the CaMV35S sequence may be responsible for *jaw-1D* paramutation, and the paramutagenic *clf-29* T-DNA could also be silenced. Indeed, the *clf-29* mutant failed to show Km resistance, although the control T-DNA insertion mutant *Atnap1;2-1* (Liu *et al.*, 2009) did (Fig. 4B). We further analysed H3K9me2 and H3K4me3 levels at several regions on T-DNA. High levels of H3K4me3 were specifically detected at *NPTII* and closely downstream of it in *Atnap1;2-1*, whereas H3K4me3 was barely detectable in any of the regions examined in *clf-29* (Fig. 4C). In contrast, H3K9me2 was detected at various levels in all regions examined in *clf-29* but was barely detectable in *Atnap1;2-1* (Fig. 4D). These data are in agreement with *NPTII* being active in *Atnap1;2-1* but silenced in *clf-29*. We then analysed DNA methylation levels within the CaMV35S region. CG and CHG methylation were at much higher levels in *clf-29* than in *Atnap1;2-1*, whereas CHH methylation was at a similar level in both mutants (Fig. 4E, Supplementary Table S2). Taken together, our data indicate that *clf-29* carries T-DNA in a heterochromatinized state containing high levels of H3K9me2 and DNA CG/CHG-methylation of CaMV35S, which is presumably the *cis*-determinant causing paramutation of *jaw-1D*.

### *jaw-1D* is responsive to silencing induced by numerous T-DNA insertion mutant lines

The *Atnap1;2-1* mutant did not show T-DNA heterochromatinization and is thus anticipated not to be paramutagenic. Indeed, our cross-test between *Atnap1;2-1* and *jaw-1D* revealed that all F1 plants had a *jaw-1D* mutant phenotype, which was in contrast to the wild-type phenotype of jaw-1D* (Table 1). The visual phenotype screen for the paramutable *jaw-1D* together with the Km-resistant screen for *NPTII* activity in T-DNA provided a powerful tool for the analysis of paramutagenic lines. We tested 19 additional T-DNA insertion mutant lines (Table 1), and first determined their Km resistance. Nine behaved like *clf-29* and displayed no resistance, one showed weak resistance, and the remaining nine showed good resistance like *Atnap1;2-1* (Table 1). We then crossed these different mutant lines with *jaw-1D* and observed the phenotype of F1 plants. Interestingly, all mutant lines displaying no or weak Km resistance could induce *jaw-1D* silencing, leading to wild-type phenotype F1 plants. In contrast, none of the Km-resistant mutant lines could induce *jaw-1D* silencing because their F1 plants showed the *jaw-1D* mutant phenotype. Hereafter, we class the first group of mutant lines as S-group (Silencing group) and the second as N-group (Non-silencing group) (Table 1).

**Table 1.** T-DNA insertion mutant lines tested in *jaw-1D* silencing

Stock number	Mutant name	Gene function	Kanamycin resistance	Silencing *jaw-1D*	Classification	CaMV35S copy number
SALK_021003	*clf-29*	*CURLY LEAF*, encoding a H3K27-methyltransferase	no	yes	S-group	16.13 ± 1.20
SALK_064627	*dcl2-1*	*DICER-LIKE 2*	no	yes	S-group	31.92 ± 1.87
SALK_065480	*sdg8-1*	*SET DOMAIN GROUP 8*, encoding a H3K27-methyltransferase	no	yes	S-group	17.62 ± 1.65
SALK_128428	*sde4-3*	*SDE4*	no	yes	S-group	27.82 ± 2.64
SALK_018714C	*mapkkk21*	*MAPKKK21*	no	yes	S-group	10.72 ± 0.84
SALK_070222	*at1g19340-1*	*Methyltransferase MT-A70 family protein*	no	yes	S-group	8.1 ± 0.62
SALK_133379	*at1g19340-2*		no	yes	S-group	22.76 ± 1.52
SALK_008485	*at1g19340-3*		no	yes	S-group	9.79 ± 0.94
SALK_152488	*chr24-1*	*CHROMATIN REMODELING 24*	no	yes	S-group	6.82 ± 0.67
SALK_149002	*atx1-2*	*ARABIDOPSIS TRITHORAX1*	no	yes	S-group	7.59 ± 0.74
SALK_069014	*chr19*	*CHROMATIN REMODELING 19*	weak	yes	S-group	12.18 ± 0.79
SALK_131746	*nap1;2-1*	*NAP1*	yes	no	N-group	2.56 ± 0.33
SALK_031553C	*ago6-2*	*ARGONAUTE 6*	yes	no	N-group	3.38 ± 0.37
SALK_064863	*hyl1-2*	*HYPONASTIC LEAVES 1*	yes	no	N-group	1.57 ± 0.16
SALK_101968C	*smc6b-1*	*STRUCTURAL MAINTENANCE OF CHROMOSOMES 6B*	yes	no	N-group	1.74 ± 0.18
SALK_047099	*arid1-1*	*AT-RICH INTERACTING DOMAIN 1*	yes	no	N-group	3.05 ± 0.33
SALK_033852	*nrpe1-12*	*NUCLEAR RNA POLYMERASE D1B*	yes	no	N-group	1 ± 0.00
SALK_020801C	*mpk17*	*MAP KINASE 17*	yes	no	N-group	1.03 ± 0.12
SALK_046208	*nrpd2a-2*	*NUCLEAR RNA POLYMERASE D2A*	yes	no	N-group	3.12 ± 0.42
SALK_039784	*hdt3*	*HISTONE DEACETYLASE 3*	yes	no	N-group	1.14 ± 0.16
SALK_127508	*at5g07810*	*SNF2 domain-containing protein*	yes	no	N-group	3.43 ± 0.36

### Tandem repeats and heterochromatinization of T-DNA probably cause *jaw-1D* silencing

**Fig. 5.**
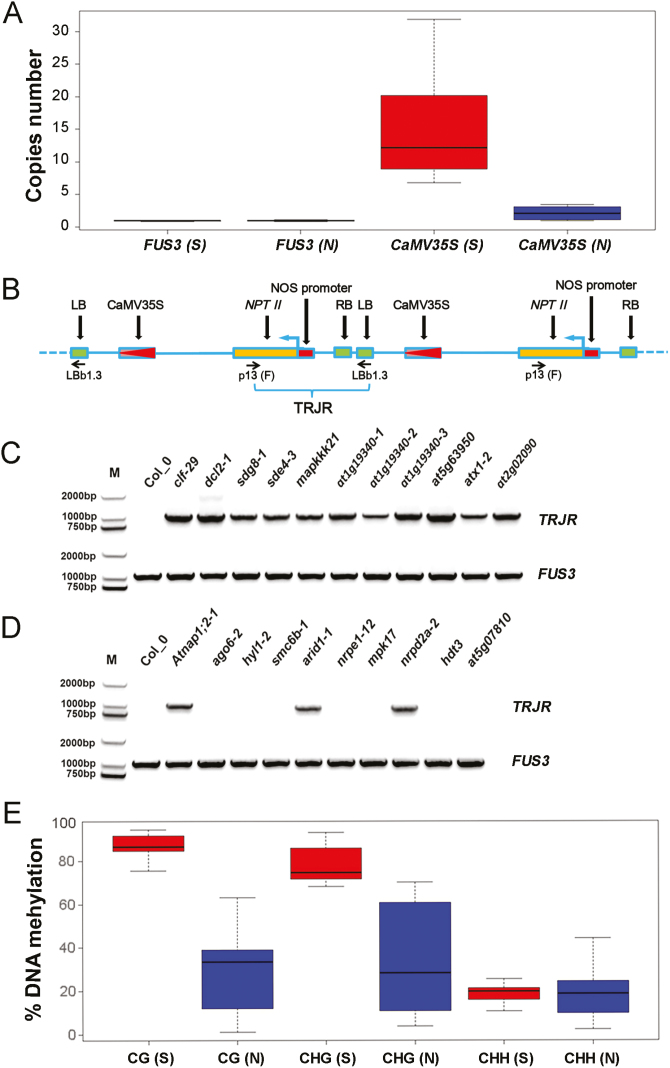
Analyses of T-DNA copy number, organization, and methylation levels in different mutant lines. (A) Box-plot showing copy number comparisons between S-group and N-group mutant lines on the endogenous control gene *FUS3* and T-DNA CaMV35S. (B) Diagram depicting the strategy used in the analysis of tandem T-DNA repeats. The T-DNA structure representation is the same as described in Fig. 4A. The PCR primers LBb1.3 and p13 (F) were designed to amplify a fragment of about 1100 bp in size only when two T-DNAs were present in tandem. TRJR, tandem repeat junction region. (C) DNA-stained gel showing PCR products amplified from S-group mutant lines as indicated. A positive TRJR signal indicates the presence of tandem T-DNA repeats and *FUS3* serves as an endogenous control. (D) DNA-stained Gel showing PCR products amplified from N-group mutant lines as indicated. Positive TRJR signal indicates the presence of tandem T-DNA repeats and *FUS3* serves as an endogenous control. (E) Box-plot showing comparisons of DNA methylation levels between S-group and N-group mutant lines. DNA methylation was analysed by bisulfite sequencing. Percentage-of-methylation values are based on >12 independent CaMV35S sequences cloned from each mutant sample. Detailed information can be found in Supplementary Table S2.

Because our tested T-DNA insertion mutant lines were all from the SALK collection that originated from transformation using the same vector (pROK2, http://signal.salk.edu/tdna_protocols.html), the T-DNA sequence alone was insufficient to explain why a line belonged to the S-group or to the N-group. During transformation, T-DNA can be inserted with a varied copy number into a site of the plant genome (Ingham *et al.*, 2001; Bubner and Baldwin, 2004). Therefore, we next examined the copy number of T-DNA in our studied mutants using qPCR. The endogenous Arabidopsis retrotransposon *TA3* was used as a normalization control. As expected, the *FUS3* endogenous gene showed no copy number difference between the S-group and N-group lines (Fig. 5A). In contrast, the CaMV35S (T-DNA) copy number varied among the mutant lines (Table 1), with S-group mutant lines showing considerably higher numbers than N-group mutant lines (Fig. 5A).

Next, we checked whether multiple copies of T-DNA were inserted as tandem repeats by using a pair of primers that would give a PCR product only if two or more T-DNA copies were present as tandem repeats (Fig. 5B). In all 11 T-DNA insertion mutant lines belonging to the S-group, we detected the PCR product indicating the presence of tandem T-DNA repeats (Fig. 5C). In contrast, among the 10 N-group T-DNA insertion mutant lines, seven showed an absence and three showed a weak signal of the PCR product for the presence of tandem T-DNA repeats (Fig. 5D). Together, these data indicated that tandem repeat multi-copies of T-DNA (CaMV35S) were associated with the induction of *jaw-1D* silencing.

Finally, we analysed DNA methylation levels at CaMV35S sequences in the S-group and N-group mutant lines. Both CG and CHG methylation were at much higher levels in S-group than in N-group mutant lines, whereas CHH methylation was at similar levels (Fig. 5E, Supplementary Table S2). These data clearly establish the crucial functions of the symmetric cytosine methylations (CG and CHG) but not the asymmetric cytosine methylation (CHH) in paramutation-like silencing.

## Discussion

In this study, we identified *jaw-1D* as a novel paramutation allele. The *jaw-1D* allele carrying the 4xCaMV35S enhancer overexpressed *miR319A* in an active chromatin mark-indexed manner (Fig. 3). It was also responsive to epigenetic silencing induced by *clf-29* as well as by many other SALK T-DNA insertion mutant lines (Table 1). Once generated, the induced silent state of *jaw-1D*, namely jaw-1D*, was not only mitotically and meiotically stable but also able to transform *de novo* the active *jaw-1D* allele into the silent jaw-1D* allele (Fig. 2). Our results further demonstrate that CaMV35S tandem repeats together with heterochromatin mark enrichments are key factors associated with *jaw-1D* paramutation (Figs 4, 5).

The 4xCaMV35S activation tagging lines are commonly used for studying gene function in Arabidopsis. When inserted upstream of a gene, the 4xCaMV35S enhancer can promote/activate its expression, leading to a gain-of-function of the gene in the mutant line. Remarkably, even when inserted downstream of a gene, such as in the case of *jaw-1D*, the 4xCaMV35S enhancer could also activate expression of the target gene. Our ChIP data revealed that it transformed the *miR319A* locus from an H3K27me3-enriched repressive chromatin state in Col-0 to an H3K4me3-enriched active chromatin state in *jaw-1D* (Fig. 3). These results are in agreement with a previous report by Chen *et al.* (2013), and together support the general notion that enhancers generate a chromatin environment that is favorable for neighboring gene transcription (Calo and Wysocka, 2013).

Despite the 4xCaMV35S enhancer containing quadruple tandem repeats of CaMV35S, the *jaw-1D* locus was stably active in *miR319A* transcription (Fig. 1), suggesting that the H3K4me3-enriched chromatin environment may also protect against silencing. However, *jaw-1D* was responsive to paramutagenic silencing by jaw-1D*. In addition, over 50% of our tested SALK T-DNA insertion mutant lines were capable of inducing *jaw-1D* silencing (Table 1). The *trans*-inactivation of CaMV35S-driven reporter genes by SALK T-DNA insertion lines has been previously observed (Daxinger *et al.*, 2008). Paramutation-like phenomena have also been described in two examples where T-DNA was inserted in the intron of an actively transcribed Arabidopsis gene. In the first example of *cob-6* where a SALK T-DNA was in the first intron of *COBRA*, the *cob-6* mutant phenotype was suppressed by crossing with the T-DNA mutant *srf6-1* or other randomly selected SALK T-DNA insertion lines (Xue *et al.*, 2012). In the second example of *ag-TD* where T-DNA was in the second intron of *AGAMOUS*, the *ag-TD* mutant phenotype was suppressed by *yuc1-1* containing the same T-DNA sequence (Gao and Zhao, 2013).

Our results extend on, and differ from, these previous studies by showing that: first, the responsive 4xCaMV35S is at the 3′-end (Fig. 1) of mi*R319A* in *jaw-1D*, so the previous finding of T-DNA location within an intron is not absolutely required; and second, that inducible lines (S-group) contain tandem repeats of CaMV35S sequences interrupted by other T-DNA sequences (Fig. 5), so paramutagenic and paramutable alleles do not need to contain the same full-length T-DNA. In maize, a hepta-repeat DNA sequence of 853 bp required for the *B-I* to *B′* paramutation is located approximately 100 kb upstream of the transcription start site of *b1* (Stam *et al.*, 2002). Collectively, it appears that *cis*-elements involved in paramutation can be in various positions or configurations in different examples of the studied genes.

Our data showing that S-group but not N-group SALK lines contain high copy numbers and tandem repeats of CaMV35S (T-DNA) are consistent with the general knowledge that paramutation is associated with DNA repeats (Hövel *et al.*, 2015). Not only was *jaw-1D* silenced in response to numerous S-group SALK T-DNA insertion lines (Table 1), but also the paramutagenic T-DNA line *yuc1-1* can induce the silencing of other different T-DNA insertion mutants such as *ag-TD* and *cob-TD* (Gao and Zhao, 2013). Nevertheless, the presence of ‘DNA repeats’ alone is insufficient to define a line as paramutagenic, as exemplified in our study by *jaw-1D* and the three N-group lines containing tandem DNA repeats that could not induce *jaw-1D* silencing. Our chromatin analyses also showed that the paramutagenic jaw-1D* and *clf-29* alleles were marked with high levels of H3K27me3, H3K9me2, and DNA CG/CHG/CHH-methylation at CaMV35S (Figs 3, 4), suggesting that repressive heterochromatinization represents a key feature for paramutagenic alleles. In support of an essential role for DNA methylation in paramutation establishment, the multi-copy *pRD29A-LUC* allele has previously been shown to be actively expressed in wild-type plants but to be converted to a silent and paramutagenic allele in *ros1*, a mutant lacking DNA glycosylase and thus accumulating a high level of DNA methylation (Zheng *et al.*, 2015).

In general, tandem DNA repeats are associated with siRNA production and are targets of DNA methylation via the RdDM pathway (Matzke and Mosher, 2014). It is highly likely that multi-copy tandem repeats of T-DNA, which occurred during *Agrobacterium*-mediated plant transformation, caused T-DNA heterochromatinization in the S-group SALK lines, and that the *trans*-interaction via homologous CaMV35S sequences between an S-group SALK line and *jaw-1D* led to jaw-1D* formation. Our study unraveled variable levels of DNA methylation of CaMV35S in different SALK T-DNA insertion mutant lines. Importantly, S-group SALK lines showed higher levels of DNA methylation than N-group SALK lines (Fig. 5E). The RdDM pathway is involved in both symmetric (CG and CHG) and asymmetric (CHH) DNA methylation (Matzke and Mosher, 2014), and components of RdDM are required in paramutation (Hövel *et al.*, 2015; Zheng *et al.*, 2015; Hollick, 2017). Symmetric CG and CHG but not asymmetric CHH within CaMV35S were found to be methylated at higher levels in S-group than N-group SALK lines, suggesting a primary role for symmetric DNA methylation in paramutation. The two methylation types are maintained by different mechanisms. CG and CHG methylations can be maintained independently of RdDM by the methyltransferases MET1 and CMT3, respectively, both of which act on hemimethylated DNA to copy the methylation from the parental strand to the daughter strand during DNA replication. In contrast, CHH methylation cannot be maintained in the absence of siRNAs, and requires re-establishment following each DNA replication cycle by the methyltransferase DRM2 (Matzke and Mosher, 2014). It is possible that both symmetric and asymmetric DNA methylations are involved in the establishment of silencing but that subsequently CG and CHG methylation is advantageous over CHH methylation in the stable maintenance of silencing, which is crucial in paramutation.

In conclusion, our study identified *jaw-1D* as a paramutable allele and highlighted the crucial functions of tandem DNA repeats and epigenetic marks in paramutation. The observed paramutation phenomena also caution against the use of *jaw-1D* in genetic interaction studies. Our results obtained from numerous T-DNA insertion lines provide a valuable and important source of information for the future investigation of gene silencing and epigenetic regulation.

## Supplementary data

Supplementary data are available at *JXB* online.

Table S1. List of primers used in this study.

Table S2. Analysis of DNA methylation of CaMV35S in different mutants.

## Supplementary Material

Supplementary_Table_S1

Supplementary_Table_S2

## Abbreviations

AGO: ARGONAUTE
AS2: ASYMMETRIC LEAVES 2
C: cytosine
clf-29: *curly leaf-29*
DCL1: DICER-LIKE 1
H3K4me3: tri-methylation of histone-3 lysine-4
H3K9me2: di-methylation of histone-3 lysine-9
H3K27me3: tri-methylation of histone-3 lysine-27
jaw-D: *jagged and wavy Dominant*
Km: kanamycin
NPTII: NEOMYCIN PHOSPHOTRANSFERASE II
PcG: polycomb group
pri-miRNA: primary miRNA
qPCR: quantitative PCR
RdDM: RNA-dependent DNA methylation
TCPs: Teosinte branched1/Cycloidea/Proliferating cell factors.
